# The Impact of Federal and State Laws on Cardiovascular Risk

**DOI:** 10.1007/s11886-025-02277-w

**Published:** 2025-08-07

**Authors:** Belinda L. Needham, Darya A. Dokshina

**Affiliations:** Department of Epidemiology, University of Michigan, 1415 Washington Heights, Ann Arbor, MI 48109 USA

**Keywords:** Legal epidemiology, Federal and state laws, Cardiovascular disease, Narrative review

## Abstract

**Purpose of Review:**

To summarize recent studies examining the impact of federal and state laws on cardiovascular disease (CVD) risk.

**Recent Findings:**

We identified 38 relevant studies that were published in the past five years. Results of the narrative review indicate that laws related to structural racism, education, income, healthcare, the food environment, food insecurity, the built environment, transportation, air pollution, tobacco, alcohol, diet, and physical activity may impact CVD risk. Results were most consistent among studies examining laws related to air pollution, tobacco, and alcohol, and least consistent among studies examining laws related to the food environment and food insecurity.

**Summary:**

Federal and state laws have the potential to shape CVD risk by impacting the social, environmental, and behavioral determinants of health.

## Introduction

Cardiovascular disease (CVD) is the leading cause of death worldwide, accounting for nearly one-third of deaths [1]. Within countries, the burden of CVD tends to be higher among socially and economically disadvantaged groups, including racial and ethnic minorities and the poor [2, 3]. Federal and state governments play an important role in shaping cardiovascular health and health disparities by enacting, implementing, and enforcing laws that impact the social, environmental, and behavioral determinants of health [4, 5]. The purpose of this narrative review is to summarize studies published in the past five years that examined the impact of federal and state laws on CVD risk. These studies are part of the growing field of legal epidemiology, which seeks to understand the role of laws in causing and preventing disease [6, 7].

### Social Determinants of CVD

**Fig. 1:**
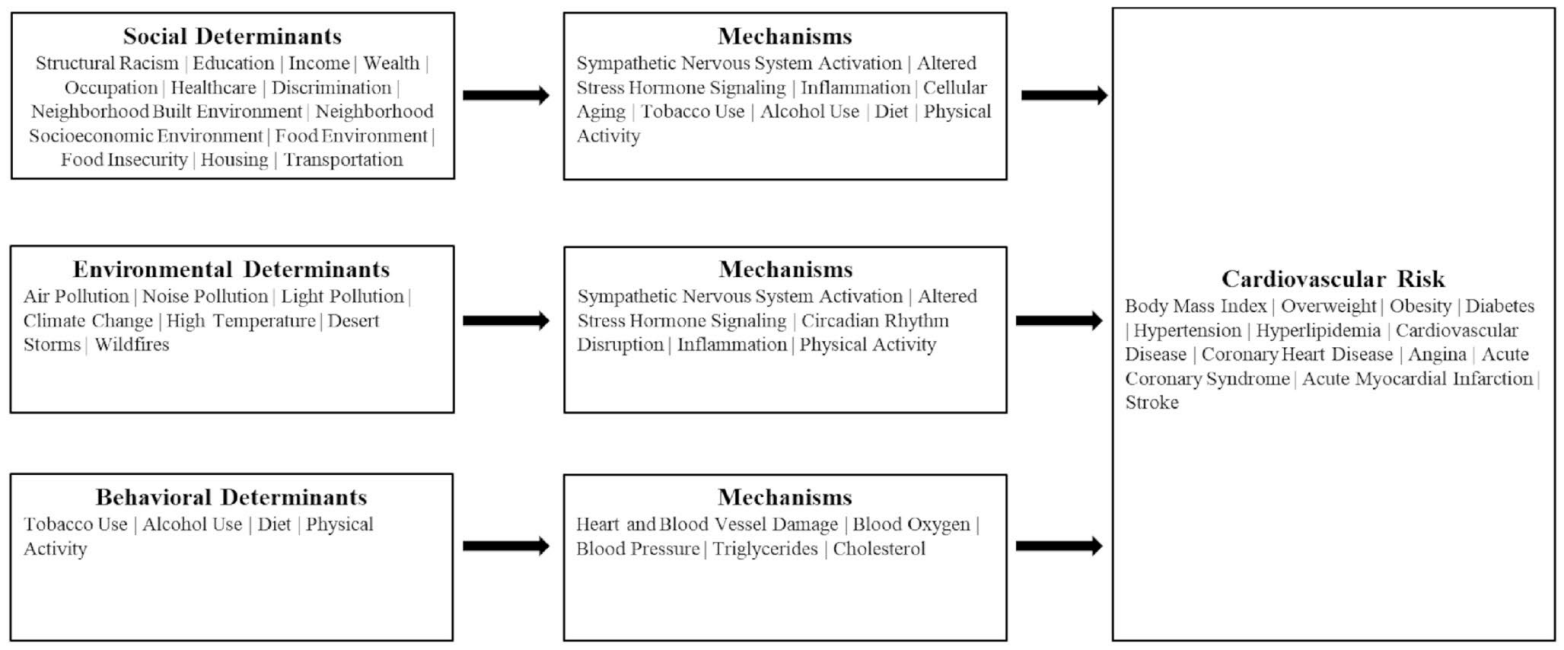
Social, environmental, and behavioral determinants of cardiovascular risk

The World Health Organization defines the social determinants of health as the conditions in which people live and work, which includes their access to material resources, such as money and housing, and their access to non-material resources, such as power and social connectedness [8]. As shown in Fig. 1, it is hypothesized that the social determinants of health influence CVD risk through stress-related behavioral and physiological pathways, such as smoking, elevated cortisol, and inflammation [9, 10]. Drawing on reviews by Powell-Wiley et al. [9] and Javed et al. [10], we identified 13 social determinants of CVD that could be impacted by federal or state laws. These include structural racism, education, income, wealth, occupation, healthcare, discrimination, neighborhood built environment, neighborhood socioeconomic environment, food environment, food insecurity, housing, and transportation. Examples of laws related to the social determinants of health include the Tax Reduction Act of 1975, which introduced the earned income tax credit in the United States; the Elliott-Larsen Civil Rights Act, which protects sexual and gender minorities from discrimination in the State of Michigan; and the American Recovery and Investment Act, which increased Supplemental Nutrition Assistance Program benefit levels.

### Environmental Determinants of CVD

A review by Munzel et al. [11] identified air pollution, noise exposure, artificial light at night, high temperatures, desert storms, and wildfires as important environmental determinants of CVD. As shown in Fig. 1, air, noise, and light pollution are hypothesized to increase CVD risk by activating the body’s stress response systems and by disrupting circadian rhythms, leading to inflammation and oxidative stress, while exposure to high temperatures is hypothesized to increase CVD risk directly and indirectly by disrupting sleep and reducing physical activity [11]. Examples of laws that impact the environmental determinants of CVD include the Ambient Air Quality Directive, which set air quality standards in the European Union, and California’s Climate Corporate Data Accountability Act, which requires businesses to disclose greenhouse gas emissions.

### Behavioral Determinants of CVD

According to the Centers for Disease Control and Prevention, health behaviors that increase CVD risk include smoking, heavy drinking, not getting enough physical activity, and eating a diet high in saturated fats, trans fat, cholesterol, and salt [12]. As shown in Fig. 1, smoking increases CVD risk by damaging the heart and blood vessels, raising blood pressure, and reducing the amount of oxygen in the blood; heavy drinking raises blood pressure and increases triglyceride levels; sedentary behavior contributes to obesity, hypertension, high cholesterol, and type 2 diabetes; and eating a diet high in saturated fats, trans fat, and cholesterol can lead to atherosclerosis, while eating too much salt can raise blood pressure [12]. Examples of laws that impact the behavioral determinants of CVD include Paraguay’s Law No. 5538, which bans indoor smoking in public places, and Singapore’s Sale of Food Act, which bans artificial partially hydrogenated oils as an ingredient in all foods sold in the country.

## Methods

**Fig. 2:**
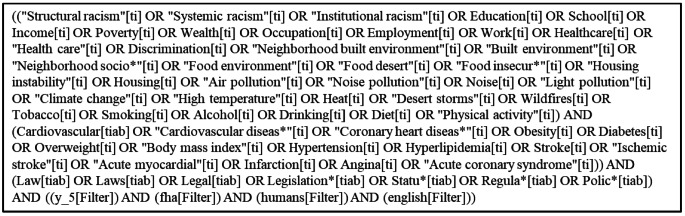
PubMed search query

We used PubMed to search for published studies examining the impact of laws on CVD risk. First, we identified terms related to the social (e.g., education, poverty), environmental (e.g., air pollution, heat), and behavioral (e.g., smoking, physical activity) determinants of CVD. Next, we identified terms related to CVD risk (e.g., coronary heart disease, stroke, obesity) and terms related to the law (e.g., legal, statute, policy). We searched for studies that included terms related to social, environmental, or behavioral determinants of CVD in the title *and* terms related to CVD risk in the title or abstract *and* terms related to the law in the title or abstract (see Fig. 2 for the PubMed search query). The search, which was conducted on May 29, 2025, and limited to human studies published in English in the past five years, identified 3,557 studies. Results from the search were transferred to Covidence for title and abstract review. We retained 43 papers after title review and 38 papers after abstract review.

## Results

### Social determinants of CVD

We identified 28 studies that examined the impact of laws related to the social determinants of CVD, including three focused on structural racism. In a study examining data from Allegheny County, Pennsylvania, Jones et al. [13] found that redlining, probable blockbusting, and urban renewal were associated with higher neighborhood-level prevalence of self-reported hypertension, coronary heart disease, and stroke, while freeway construction was associated with lower prevalence of hypertension and coronary heart disease. In a quasi-experimental study examining the impact of school segregation on cardiovascular health among Black adults in the Panel Study of Income Dynamics (PSID), Kim et al. [14] found that segregation was not associated with heart disease (although results were statistically significant in standard linear models). Using data from the Behavioral Risk Factor Surveillance System (BRFSS), Needham et al. [15] found that an increase in the structural racism state legal index, which included 22 structural racism-related state laws in the domains of criminal justice, economics and labor, education, healthcare, housing, immigration, and political participation, was associated with a decrease in the Life’s Simple 7 cardiovascular health score, with no significant differences by race and ethnicity.

With respect to individual-level measures of socioeconomic status, we found five studies that examined laws related to education, one study that examined laws related to income, and no studies that examined laws related to wealth or occupation. A quasi-experimental study by Rizal et al. [16] examining the long-term health impacts of Indonesia’s primary school construction initiative in the 1970s found that the program was associated with increased likelihood of overweight and high waist circumference among women but not among men. Another quasi-experimental study by Liu et al. [17] examining the impact of China’s nine-year compulsory education law found that each additional year of schooling for adult children was associated with a 5% reduction in the prevalence of hypertension among parents with no effect on the prevalence of diabetes. In a study of high school students in Mississippi, Jang et al. [18] found that average body mass index (BMI) decreased after implementation of school-based policies that emphasized physical activity, physical education, nutrition standards, and nutrition education. A quasi-experimental study using data from the Early Childhood Longitudinal Studies found that state laws mandating physical education and physical activity time in elementary schools did not lead to significant increases in physical activity in schools or to any changes in BMI or prevalence of overweight or obesity [19]. In a study of Oregon schools, Tomayko et al. [20] found that schools with four-day school weeks offered more physical education than five-day schools, but there was no significant difference in obesity prevalence between the two types of schools. Finally, a study examining the impact of an income redistribution policy in New Zealand found that implementation of the policy led to a 0.98% reduction in the prevalence of obesity among women [21].

We identified five studies that examined laws related to healthcare. In a quasi-experimental study using data from BRFSS, Engel Rebitzer et al. [22] found that adults in Arkansas with cardiovascular risk factors experienced a non-significant decrease, relative to adults in control states, in health insurance coverage after implementation of a Medicaid work requirement. Using a similar design, Gotanda et al. [23] found that individuals in Medicaid expansion states experienced a significant reduction in systolic blood pressure and improvement in HbA1c compared to those in control states. While one study of low-income nonelderly adults found a 38.1% greater increase in the rate of Medicaid beneficiaries with outpatient visits for CVD management and a 42.9% greater increase in the prescription rate for drugs to manage CVD in expansion states compared to control states [24], another study found no significant differences in treatment for cardiovascular risk factors between Medicaid expansion states compared to control states [25]. A study in China found that the proportion of individuals receiving education, undergoing examination, and receiving treatment for diabetes improved following implementation of a program designed to reduce geographic disparities in access to care, although results were weaker than expected [26].

Five of the 11 studies that examined laws related to the food environment or food insecurity were reviews or meta-analyses. A systematic review by Cohen et al. [27] found no association of universal free school meal programs with increased BMI among students in developed countries. However, there was some evidence that these programs might reduce obesity if they met nutritional guidelines [27]. A review by Gallegos et al. [28] found that school-provided meal programs in high-income countries offer nutritional benefits but are not consistently associated with BMI, while a meta-analysis by Liu et al. [29] found that nutrition policies, such as school nutrition programs, reduce childhood obesity risk in high-income countries but not in low- or middle-income countries. A systematic review by Olarte and colleagues [30] reported that some studies examining the impact of alternative school breakfast service models found that these programs were associated with increased BMI; however, results were not consistent across studies. Finally, a systematic review of studies evaluating the impact of taxes on sugar-sweetened beverages reported that most studies found that higher prices achieved through taxation were associated with lower prevalence of overweight and obesity across countries with varying income levels [31].

Six original empirical studies also examined laws related to the food environment or food insecurity. A quasi-experimental study evaluating the impact of California’s competitive food and beverage policies, which regulate the nutritional content of foods and beverages for sale in schools, found that children in suburban areas showed the greatest reductions in obesity after the policy was introduced, with some beneficial impacts in rural and second city areas and in urban areas [32]. In another quasi-experimental study, Localio et al. [33] found that California schools that adopted the community eligibility provision of the federal Healthy, Hunger-Free Kids Act (HHFKA) showed a decrease in obesity prevalence compared to eligible non-participating schools. A similar study by Matsuzaki et al. [34] found that increases in obesity slowed in California after the introduction of the HHFKA and competitive food and beverage policies, especially among White and Filipino boys. Consistent with these results, a study by Sanchez-Vaznaugh et al. [35] found that child overweight and obesity rates stabilized or went down in California following the introduction of state school nutrition policies, with further improvements seen after the HHFKA was passed. A quasi-experimental study in Norway found no significant impact of a free fruit and vegetable policy on weight-related outcomes among children [36]. And in a study of European Union member states, Lovas et al. [37] found null or weak correlations between nutritional policies and prevalence of diabetes.

We found two studies that examined laws related to the built environment and one study that examined transportation policy. An agent-based model simulating implementation of a Safe Routes to School (SRTS) program in El Paso, Texas (part of the federal Safe, Accountable, Flexible, Efficient Transportation Act) projected 157 fewer cases of heart disease and 217 fewer stroke cases per 10,000 people when SRTS policies are in place [38]. Lovas et al. [37] found null or weak correlations between physical activity policies and prevalence of diabetes in European Union member states, and Choi et al. [39] found that introduction of a taxi service for elderly patients with diabetes in Korea was associated with an increase in outpatient visits to public health centers, an increase in visits to general hospitals, and a decrease in visits to tertiary hospitals. We did not find any studies examining laws related to interpersonal discrimination, neighborhood socioeconomic status, or housing instability.

### Environmental determinants of CVD

We identified two studies that examined laws related to air pollution. A quasi-experimental study by Fan et al. examining the effect of China’s Two-Control Zone environmental policy found that a reduction of 1 µg/m^3^ of sulfur dioxide was associated with a 0.9% decrease in cardiovascular deaths per 100,000 people aged 60+ [40]. Another quasi-experimental study in Weifang, China found that acute myocardial infarction rates decreased by 6.5% after implementation of air pollution control policies, with larger effects on women and the elderly [41]. We did not find any studies examining laws related to noise or light pollution, high temperature, desert storms, or wildfires.

### Behavioral determinants of CVD

We identified 25 studies that examined laws related to behavioral determinants of CVD, including seven related to tobacco. A quasi-experimental study by Montes de Oca et al. [42] found a decrease in hospitalization and mortality rates for ischemic heart disease, acute myocardial infarction, and stroke following Chile’s indoor smoking ban, with larger effects in younger and middle-aged groups. In an evaluation of Vietnam’s Law on Prevention and Control of Tobacco Harms, Nguyen et al. [43] found that raising tobacco taxes was the most effective intervention for minimizing potential disability-adjusted life years due to CVD, while enforcing bans on advertising, promotions, and sponsorship of tobacco products was the least effective. Patanavanich et al. [44] found that an increase in cigarette taxes in Thailand led to a 4.7% reduction in acute myocardial infarction hospitalizations among adults under 45, while a 100% smoke-free law led to a 13.1% reduction in such hospitalizations. In a study of male prisoners who were smokers before entering prison, Perrett et al. [45] found that a smoking ban implemented in prisons across England and Wales resulted in a decrease in predicted age to CVD events and heart age. Quitting smoking decreased the chances of having a stroke or myocardial infarction by 5% over the next 10 years for male prisoners aged 50 + who were heavy smokers [45]. A simulation study by Salgado et al. [46] estimating the impact of plain packaging for tobacco products in Argentina found that an expected 0.55% decrease in smoking prevalence was projected to result in 1,880 fewer myocardial infarctions and 820 fewer strokes. A quasi-experimental study by Wu et al. [47] found that Beijing’s comprehensive tobacco control policies, including an indoor smoking ban, increased taxes, cessation support, and a ban on advertising, led to a 13.4% decrease in hospital admissions for CVDs during the 25 months following implementation. In a systematic review and meta-analysis of 144 population-level studies examining the health impact of smoke-free legislation, tax and price increases, and multicomponent tobacco programs, Akter et al. [48] found that smoke-free legislation was associated with a 9–10% reduction in the odds of overall CVD events and a 9% reduction in hospitalizations related to CVDs.

We found two studies that examined laws related to alcohol. In an ecologic study of 169 countries, Diaz et al. [49] found that a higher score on the alcohol preparedness index (based on policies such as taxes, pricing, advertising, and drunk driving penalties) was associated with lower CVD prevalence and mortality. A quasi-experimental study by Kim et al. [50] examining the impact of three Lithuanian alcohol control policies, including price increases, reduced availability, or both, on sex- and stroke subtype-specific mortality rates found that the policies had the greatest impact on ischemic stroke mortality rates among women.

Each of the 12 studies that examined laws related to diet [18, 27–31, 33–37] were also related to the food environment or food insecurity and are summarized above. Similarly, each of the five studies [18–20, 37, 38] that examined laws related to physical activity were also related to education or the built environment and are summarized above.

## Discussion

This narrative review summarized 38 recent studies examining the impact of federal and state laws on CVD risk. Studies examined a wide variety of laws related to the social, environmental, and behavioral determinants of CVD, including laws related to residential segregation, compulsory education, income redistribution, healthcare access, neighborhood walkability, school nutrition, air pollution, tobacco packaging, and the cost of alcohol and sugar-sweetened beverages. Studies also examined a wide variety of cardiovascular-related outcomes, including obesity, hypertension, diabetes, coronary heart disease, acute myocardial infarction, and stroke. Results were most consistent among studies examining laws related to air pollution, tobacco, and alcohol, and least consistent among studies examining laws related to the food environment and food insecurity.

Despite the variety of laws and cardiovascular outcomes examined in recent studies, some appeared more frequently than others. For example, nearly one-third of the studies included in this review examined the impact of laws regulating the food environment and diet on weight-related outcomes, with most of these studies focused on school nutrition programs for children. Studies examining tobacco-control policies and laws related to education, healthcare, and physical activity were also common, while studies examining laws related to income, wealth, employment, discrimination, housing, transportation, noise and light pollution, and climate were limited. Future studies should examine how universal basic income programs, tax policies, income assistance programs, occupational safety laws, non-discrimination laws, housing assistance programs, public transportation policies, policies that regulate noise emissions and outdoor lighting, laws that improve access to indoor cooling, and disaster response laws impact CVD risk. In addition, future studies should consider whether these and other laws impact racial, ethnic, socioeconomic, and geographic disparities in CVD risk.

A limitation of this review is that our search strategy required that studies include one or more legal terms, such as law, legislation, statute, regulation, or policy, in the title or abstract. This approach produced a manageable number of abstracts to review but likely missed relevant studies. For example, studies that referred to the Affordable Care Act in the title or abstract did not appear in our search unless they also included a legal term in the title or abstract. Although PubMed includes a medical subject heading (MeSH) for legal epidemiology [7], only 12 studies have been indexed using this MeSH term over the past five years, and none were focused on cardiovascular outcomes. Relevant studies will become easier to identify as researchers begin to explicitly situate their work within the field of legal epidemiology.

## Conclusions

Governments play a crucial role in shaping cardiovascular health by enacting, implementing, and enforcing laws related to the social, environmental, and behavioral determinants of health [4, 5]. This review highlighted examples of recent studies examining the impact of federal and state laws on CVD risk, calling attention to the importance of a Heath in All Policies approach [51]. Future studies in this area should continue to employ quasi-experimental designs, which strengthen causal inference [7], and expand the use of complex system approaches, which can be used to explore the effects of policy interventions, taking into account dynamic relations between multi-level causes of disease [52, 53]. Given that the burden of CVD tends to be higher among socially and economically disadvantaged groups, it is also important for future studies to consider whether federal and state laws reduce or exacerbate cardiovascular health disparities.

### Key References

- Burris S, Cloud LK, Penn M. The Growing Field of Legal Epidemiology. J Public Health Manag Pract. 2020;26:S4–9.
-
  - **Presents an overview of the field of legal epidemiology.**
- Leppo K, Vereinte Nationen, editors. Health in all policies: seizing opportunities, implementing policies. s.l: Ministry of Social Affairs and Health, Finland; 2013.
-
  - **Discusses the importance of considering the health implications of policies across all sectors, not just those directly related to health or healthcare.**

## Data Availability

No datasets were generated or analysed during the current study.
